# Transnasal sphenopalatine ganglion block for pain relief during panretinal photocoagulation laser for diabetic retinopathy: a pre and post interventional study

**DOI:** 10.1038/s41598-022-14745-2

**Published:** 2022-06-29

**Authors:** Mehdi Sanatkar, Fatemeh Bazvand

**Affiliations:** 1 Pain Research Center, Neuroscience Institute, Tehran University of Medical Sciences, Tehran, Iran; 2 Eye research center, vitreoretinal department, Farabi Eye Hospital, Tehran University of Medical Sciences, Tehran, Iran; 3 Department of Anesthesiology and Critical Care, Imam Khomeini Medical Center, Tehran university of medical science, Tehran, Iran

**Keywords:** Medical research, Outcomes research

## Abstract

This study was performed to utilize transnasal Sphenopalatine Ganglion (SPG) block for pain reliving during panretinal photocoagulation (PRP) in diabetic patients with diabetic retinopathy. This pre and post interventional study was performed on 20 patients with proliferative diabetic retinopathy. The first PRP treatment session of all the patients is performed with no transnasal SPG block, but before holding the second session, all the patients underwent transnasal SPG block and pain levels during and after PRP were compared to each other. Before the transnasal SPG block, each nostril of all the cases was inspected for finding any obstruction in each PRP session. Transnasal SPG block was also performed in with 2% lidocaine. The mean age of the included cases was 52.84 ± 8.62 years old (from 36 to 72 years old). All the cases underwent two PRP lasers treatment sessions with the same characteristic (spot size, power and duration) for each patient. In the first and second PRP treatment sessions, the mean NRS scores were obtained immediately after the PRP laser (8.4 vs. 4.2), 15 min (8.2 vs. 4.2), 1 h (8.0 vs. 4.1), and 24 h (5.4 vs. 3.6) after the PRP respectively. The mean NRS scores significantly reduced during the second PRP treatment session compared to the first session (p < 0.001). Transnasal SPG block is a safe and effective strategy used for relieving pain caused by the PRP laser treatment in patients with diabetic retinopathy.

## Introduction

Diabetic retinopathy is known as the most important cause of visual loss in diabetic patients. The most acceptable treatment of visual impairment in diabetic patients caused by proliferative diabetic retinopathy, is the administration of panretinal photocoagulation (PRP) laser^1,2^, which destroys the ischemic areas on the retina. Moreover, a study on the diabetic retinopathy identified that PRP with the placement of 800–1600 burns with 500-microns diameter and 0.1 s duration could induce at least 50% reduction in visual impartment within a 5-year period^3^. The main reason for the decreased burning delivered in these patients is pain during PRP laser^4,5^. Most of the cases undergoing PRP treatment complain of moderate to severe level of ocular pain during the procedure. In this regard, in previous studies, many authors have tried different strategies such as oral acetaminophen or intramuscular ketorolac, oral mephenamic acid, oral diazepam, oral or topical diclofenac, sub-tenon anesthesia, and peribulbar anesthesia for relieving pain during PRP, but there are no good evidences of the efficacy for any of them yet^6–11^. Moreover, other strategies such as transcutaneous electrical nerve stimulation^12^ and Entonox (50:50 nitrous oxide: oxygen gas) have limited its effects because of the need either for special equipment or invasiveness, or both of them. The sphenopalatine ganglion (SPG), also known as ptyergopalatine ganglion, is a large extracranial parasympathetic ganglion with multiple neural connections, including autonomic and sensory^13^. Accordingly, it is located in the upper part of the sphenomaxillary fossa in posterior to the middle nasal turbinate, from radiated nerves fibers to eyes. This ganglion is involved in pain processing through facial and trigeminal nerves as well as cervical sympathetic chain. Therefore, we thought that maybe we can reduce the pain during the PRP process with the SPG block. The history of SPG block dates back to when Sluder has reported that SPG block with cocaine could help in the treatment of headache and facial pain syndromes^14^. After a while, SPG block has gained interest as an effective strategy for management of headache such as cluster or migraine and facial pain syndrome^15^. Transnasal SPG block is an easy, effective, and safe method used for the treatment of acute migraine headache. Because of this ganglion’s involvement in pain processing of both face and eyes, in the current study, we aimed to use transnasal SPG block for pain reliving during PRP in diabetic patients with diabetic retinopathy.

## Materials and methods

This pre and post interventional study was performed to evaluate the efficacy of transnasal SPG block in pain relief during performing PRP laser for diabetic retinopathy among diabetic patients. Our study was conducted at Farabi hospital affiliated to Tehran University of medical sciences, Tehran, Iran, from March 2021 to September 2021. Moreover, it was approved by the ethical committee of our hospital and performed in terms of the tents of the Helsinki. All the included patients signed the informed consent form. The informed consent for publication of figure also was obtained. The inclusion criteria were the followings: diabetes diagnosed after the age of 30 years old, proliferative diabetic retinopathy, no history of PRP, intraocular pressure less than 21 mmHg, and clear vitreous (media). In addition, the exclusion criteria were the followings: previous PRP, cataract, vitreous hemorrhage, bleeding tendency, concomitant neovascular glaucoma, history of any psychiatric problem, severe abnormal neurological examination, the chronic use of analgesics, and history of allergy to local anesthetic. All the included patients underwent the complete ophthalmological examinations such as fundoscopy, tonometry, gonioscopy, bio-microscopy, and best-corrected visual acuity before performing PRP. As well, after the first session of the PRP treatment, they completed the pain sensation assessment scored based on a numeric rating scale (NRS), where 0 indicates no pain, and 10 is the worst pain imaginable. In order to perform papillary dilation, Mydrax 0.5% was administered 30 min before the PRP, for 3 times in each eye of all the patients. In case of any inadequate pupillary dilation, repeating the administration of dilating drop was allowed. Notably, the PRP treatments were performed only by one experienced retinal specialist (FB). The PRP was conducted using Argon green laser with a panretinal contact lens (superquad). Each session of PRP was done using the green laser (wavelength: 514) with spot size of 500 µm, in a duration of 0.2 s (200 ms), and power with a moderate intensity level (200–500 mW). A total of 1500–1800 spots were performed for each eye.

**Figure 1:**
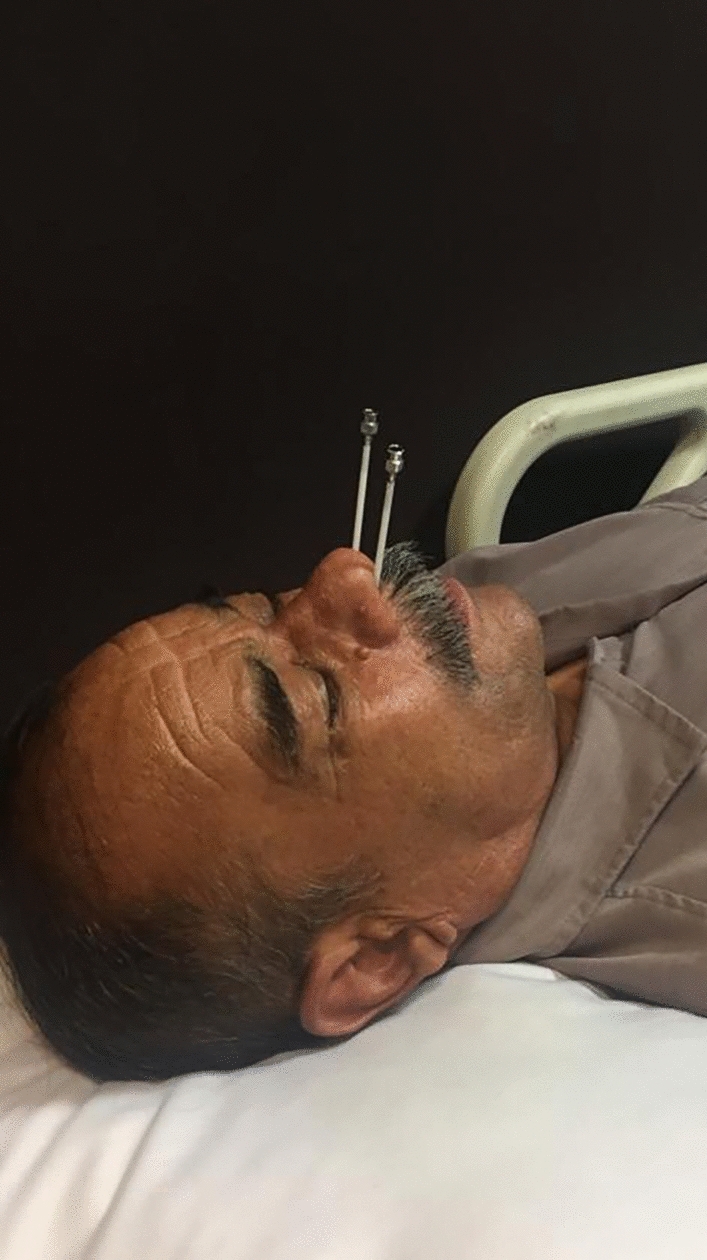
The method of transnasal sphenopalatine ganglion block for pain relief during laser.

The first PRP treatment session (the inferior half of retina with spare horizontal meridians) of all the patients is performed with no transnasal SPG block, but before holding the second session of the laser treatment (the superior half of retina with spare horizontal meridians), all the cases underwent transnasal SPG block and pain levels during and after PRP were compared to each other. Before the transnasal SPG block, each nostril of all the cases was inspected for finding any obstruction in each PRP session. For patients’ comfort during this procedure, the anterior of each nostril was anesthetized with 4% lidocaine aerosol spray. Transnasal SPG block was also performed in each patient with 2% lidocaine in each nostril in supine position with head extension. To further reduce the patients’ discomfort, this procedure was conducted using a cannula in order to deliver lidocaine instead of cotton-tipped applicators. In this regard, a cannula preloaded with 2 ml of 2% lidocaine was inserted into the nostril along the upper border of the inferior turbinated directed backwards until reaching the upper posterior wall of the nasopharynx. Thereafter, 1 ml lidocaine 2% each at 5 min interval were administered for a 20-min duration (Fig. 1). All the studied cases were monitored in terms of their vital signs during the procedure. Moreover, the patients were asked to stay in the same position for 10 min and then underwent PRP laser. Immediately after the PRP laser; and 15 min, one, and 24 h after the completion of the PRP laser in both laser sessions, with or without transnasal SPG block, all the subjects were asked to rate their level of pain on NRS. Furthermore, they were evaluated in terms of any ocular or systemic side effects. The range of laser setting used in the second PRP laser treatment was similar to that of the first session. Of note, no subject received sedation during the PRP laser treatment.

### Statistical analysis

In the present study, the main purpose was to evaluate the efficacy of transnasal SPG block in pain reduction during and after the PRP laser treatment in the first session without using any SPG block compared to the second session using SPG block. The secondary outcome measures were ocular and systemic side effects in both the first and second PRP laser treatment sessions. Based on the pain scores, and detecting a least a 3-point difference between scores and an estimated standard deviation of 3.8 as well as providing a power of 80% with a 2-tailed significant level of 0.05, 20 patients were finally enrolled in our study. The normal distribution of data was documented by Shapiro–Wilk test. A Chi-square test or Fisher’s exact test was then used to compare the discrete variables (like headache, nausea, and etc.) and Student’s paired t-test was also applied for continues variables. The statistical significance level was considered at p < 0.05.

### Ethics approval and consent to participate

The patients provided written informed consent. The institutional review board/ethics committee of the Tehran University of Medical Science approved this survey.

### Consent for publication

Written informed consents were obtained from patients.

## Results

A total of 20 patients with both proliferative diabetic retinopathy who were candidates for PRP laser treatment, were enrolled in the current study from April to June 2021. Accordingly, their characteristics are summarized in Table 1. The mean age of the included cases was 52.84 ± 8.62 years old (from 36 to 72 years old). All the cases underwent two PRP lasers treatment sessions. The first session was done without any blockage and the second session was done with blockage. The patients were evaluated for the level of pain during and after the procedure in both sessions. The range of elapsed time between the two PRP treatment sessions was 1 to 2 weeks. All the patients were suffering from proliferative diabetic retinopathy (PDR). The spot size (500 µm), power (range 250–500, same power was applied for each patient during these 2 sessions), duration (0.2 s), and laser spot number (750–900) were similar in both sessions. In the first PRP treatment session, the mean NRS scores calculated immediately after the PRP laser, 15 min, 1 h, and 24 h after the PRP laser were obtained as 8.4, 8.2, 8.0, and 5.4, respectively. While in the second PRP treatment session using transnasal SPG block, the mean NRS scores calculated immediately after the PRP laser, 15 min, 1 h and 24 h after the PRP laser were obtained as 4.2, 4.2, 4.1, and 3.6, respectively. The mean NRS scores significantly decreased during the second PRP laser treatment compared to the first PRP laser treatment (p < 0.001). Overall, the procedure was well-tolerated during the PRP treatment sessions. As well, the side effects caused by each PRP laser treatment session^8^ are shown in Table 2. Of note, local anesthetic toxicity, epistaxis, and intranasal trauma have not been reported. In the present study, the patients were monitored for 30 min and none of the patients had any serious side effects.

**Table 1:** Baseline demographic and clinical characteristics of patients.

Variable		
Number of patients		20
Demographic data	Age (years, mean ± SD)	52.84 ± 8.62
	Gender (M/F)	12/8
	DM type (IDDM/NIDDM)	4/16
Past medical history	Duration of diabetes (years)	3.8–12.6
	Hypertension, n (%)	12 (60%)
	Diabetic neuropathy, n (%)	16 (80%)
	Cardiovascular disease, n (%)	10 (50%)
	Previous history of stroke without any sequel, n (%)	2 (10%)
Clinical findings	Proliferative diabetic retinopathy or PDR (early vs. high risk PDR)	20 (11/9)
	Intraocular pressure	15.6 ± 4.2

*DM* diabetes mellitus, *IDDM* insulin dependent diabetes mellitus, *NIDDM* non insulin dependent diabetes mellitus.

**Table 2:** Side effects of the panretinal photocoagulation^8^ in two sessions.

Side effect	1st session of PRP	2nd session of PRP
Headache, n	12	5 (p = 0.21)*
Dizziness, n	6	2 (p = 0.13)*
Photophobia, n	5	0
Nausea, n	4	0
Eye itchiness, n	3	0
Eye pain, n	16	5 (p = 0.1)*
Agitated/restless, n	4	0

*PRP* panretinal photocoagulation.

*p values represent statistical analysis with the Fisher exact test of the differences in proportions between 1st session of PRP and 2nd session of PRP.

## Discussion

Proliferative and non-proliferative diabetic retinopathies are known as one of the major causes of visual disturbance in diabetic patients. Additionally, it was shown that the PRP laser treatment is an effective strategy on decreasing the rate of visual loss in these patients. However, the PRP laser is a painful treatment for these cases, and the pain of laser intolerance leads most patients not to complete their laser procedure^16^. Therefore, it is necessary to decrease the pain during this procedure and increase tolerance level in patients who need PRP laser treatment. This study was designed due to relatively much pain during and after performing the laser that was shown to be able to affect the cooperation of patients. To the best of our knowledge, this study was the first aimed to present transnasal SPG block for decreasing PRP laser pain in patients. This study showed that transnasal SPG block is a safe, minimally invasive, and effective method used for pain relief during and after the PRP laser treatment in patients with diabetic retinopathy. The current research observed the decreased PRP laser pain during laser, 15 min, 1 h, and 24 h after performing the procedure in the studied cases. In this regard, the NRS scores decreased from 8.4 to 4.2 immediately after the PRP laser treatment. Moreover, in previous studies, no safe and effective agent or strategy could reduce laser pain in these patients. In previous studies, it was identified that acetaminophen, diazepam, mefenamic acid, and intramuscular ketorolac are not effective on pain reliving of PRP laser^7,9^. Moreover, some authors have employed numerous invasive strategies for reliving PRP pain such as sub-tenon anesthesia, and retrobulbar anesthesia, but these methods were indicated to possibly increase the risk of the potential events such as conjunctival and eyelid edema, akinesia of the eyelid, orbital hematoma, and globe perforation, so they are not suitable for use in offices^17,18^.

Other strategies for reduction this pain were alternations in laser parameters. In a study that compared the level of pain of short laser exposure (0.02 s) with that of traditional method (0.1 s), statistically significant less pain was found in the first group^19^. Another study has evaluated different diode laser pulse shapes and showed that the severity of pain with standard square-wave mode was more than the altered shaped wave and micropulse modes^20^. Finding an easy, non-invasive, and safe strategy for decreasing the pain level during the PRP laser treatment leads patients to better tolerate laser treatment, and most patients benefit from the PRP outcomes. The results of our study show that transnasal SPG block could provide statistically significant pain reduction both during and after PRP laser treatment. Some previous studies have concluded that the pain level of the second PRP treatment was significantly more than that of the first PRP laser session^21^. This difference might be resulted from the central sensitization of pain during the second PRP laser treatment. Therefore, when pain in the second PRP laser treatment was observed to be lower than the first session by using transnasal SPG block, this reflects the suitable effect of this block on controlling the pain in these patients.

Accordingly, the question that arises is how can the transnasal SPG block help in relieving pain during the PRP laser treatment? Many authors studied the mechanism of ocular pain during PRP laser. Previous studies have shown that migraine is triggered by the stimulation of trigemino-autonomic reflex, which is related to the increased parasympathetic outflow from SPG. SPG block can also help in pain relief of a migraine attack. SPG blocks cause reduction of neuroinflammatory mediators from sensory nerves such as substance P, acetylcholine, nitric oxide and calcitonin gene-related peptide by the inhibition of parasympathetic outflow and consequently led to the decreased intensity of pain and central sensitization^22^. Therefore, we thought that using SPG block could help to reduce pain during PRP laser through the suppression of trigemino-autonomic reflex. Moreover, another mechanism explaining the possible effect of SPG block in decreasing pain through trigeminal nucleus was the afferent sensory fibers, which may reduce pain by reducing central sensitization^23,24^. The patients complained about having severe pain in and around their eyeball during PRP laser. Additionally, many patients’ complaints were related to radiate pain to the ipsilateral upper lid and some areas of the head such as forehead, temporal, and parietal during PRP laser. Of note, the sensory pathway of ocular pain is transmitted by the long ciliary nerve through nasociliary nerve and merging with the ophthalmic nerve^25^. It was shown that the ophthalmic nerve, which is the first branch of the trigeminal nerve, was stimulated during the PRP laser and then caused a painful sensation in the patients. Besides experiencing intraocular pain during the PRP laser, the patients often complained about pain around the eyes, which is related to pain sensation by the supraorbital, supratrochlear, and lacrimal nerves, all of which belong to the ophthalmic nerve^26^. Furthermore, it was shown that the pain during the PRP laser treatment is caused by the photocoagulation of long and short ciliary nerves running in the suprachoroidal space^27^. It is noteworthy that Ciliary ganglion includes sympathetic, parasympathetic, and sensory fibers. Therefore, it is expected that SPG block can help in reducing the PRP laser pain through sympathetic, parasympathetic, and sensory blocks. The sympathetic originates in the superior cervical ganglion around the internal carotid artery, which joins the greater petrosal nerve and then both form the Vidian nerve, which enters the SPG^13,28,29^. The parasympathetic preganglionic cell bodies originate in the superior salivatory nucleus in the pons, and in this way, parasympathetic nerves run in the nervus intermedius, form greater petrosal nerve, and finally enter SPG^15,30,31^. As well, the sensory fibers that carry sensation from tonsil, buccal, and palate through the maxillary nerve, enter SPG^22,28,32^. Therefore, the SPG can be considered as a special position that is involved in pain and central sensitization through multiple neural connections of sympathetic, parasympathetic, and sensory fibers. Based on previous findings, it was thought that transnasal SPG block could help to reduce PRP laser pain, which is not considered as an effective method to control their pain, and our study finally confirmed this hypothesis.

Our study has some limitations. The main limitation of our study was our small sample size. The other limitation of our study is that, in some patients may experience less discomfort after the first session and it may be influence our results. However, the significant differences were seen between 2 sessions and it can partly compensate some bias. In this study, we selected bilateral cases that never had PRP laser treatment before, in order to reduce any kind of bias. More well-designed studies like clinical trials are needed to evaluate the efficacy of transnasal SPG block in reducing pain during the PRP laser treatment.

Finally, it can be concluded that transnasal SPG block is an easy, safe, and effective strategy used for relieving pain caused by the PRP laser treatment in patients with diabetic retinopathy. Some advantages such as its ease of administration, safety profile, and effective pain relief make this method an attractive treatment option for patients who cannot benefit from PRP laser treatment due to having pain. However further studies with well-designed and more cases are required for the efficacy of SPG block.

## Data Availability

The datasets used in the current study are available upon reasonable request.

## Abbreviations

PRP: Panretinal photocoagulation
SPG: Sphenopalatine ganglion
NRS: Numeric rating scale

## References

[1] Fullerton B, Jeitler K, Seitz M, Horvath K, Berghold A, Siebenhofer A (2014). Intensive glucose control versus conventional glucose control for type 1 diabetes mellitus. Cochrane Database Syst. Rev..

[2] Chew EY, Ferris FL, Csaky KG, Murphy RP, Agrón E, Thompson DJ, Reed GF, Schachat AP (2003). The long-term effects of laser photocoagulation treatment in patients with diabetic retinopathy: The early treatment diabetic retinopathy follow-up study. Ophthalmology.

[3] Preliminary report on effects of photocoagulation therapy. The Diabetic Retinopathy Study Research Group. *Am. J. Ophthalmol.* **81**, 383–396 (1976). 10.1016/0002-9394(76)90292-0 944535

[4] Dowler JG (2003). Laser management of diabetic retinopathy. J. R. Soc. Med..

[5] Vaideanu D, Taylor P, McAndrew P, Hildreth A, Deady JP, Steel DH (2006). Double masked randomised controlled trial to assess the effectiveness of paracetamol in reducing pain in panretinal photocoagulation. Br J Ophthalmol..

[6] Wu WC, Hsu KH, Chen TL, Hwang YS, Lin KK, Li LM, Shih CP, Lai CC (2006). Interventions for relieving pain associated with panretinal photocoagulation: A prospective randomized trial. Eye (Lond.).

[7] Al-Hussainy S, Dodson PM, Gibson JM (2008). Pain response and follow-up of patients undergoing panretinal laser photocoagulation with reduced exposure times. Eye (Lond.).

[8] Zakrzewski PA, O'Donnell HL, Lam WC (2009). Oral versus topical diclofenac for pain prevention during panretinal photocoagulation. Ophthalmology.

[9] de Araújo RB, Zacharias LC, de Azevedo BM, Giusti BS, Pretti RC, Takahashi WY, Monteiro MLR (2015). Metamizole versus placebo for panretinal photocoagulation pain control: A prospective double-masked randomized controlled study. Int. J. Retina Vitreous.

[10] Tesha PE, Giavedoni LR, Berger AR, Altomare F, Chow DR, Navajas EV, Yoganathan P, Wong DT, Principe A (2010). Subconjunctival lidocaine before laser treatment: A randomized trial. Ophthalmology.

[11] Weinberger D, Ron Y, Lichter H, Rosenblat I, Axer-Siegel R, Yassur Y (2000). Analgesic effect of topical sodium diclofenac 0.1% drops during retinal laser photocoagulation. Br. J. Ophthalmol..

[12] Whitacre MM (1991). The effect of transcutaneous electrical nerve stimulation on ocular pain. Ophthalmic Surg..

[13] Piagkou M, Demesticha T, Troupis T, Vlasis K, Skandalakis P, Makri A, Mazarakis A, Lappas D, Piagkos G, Johnson EO (2012). The pterygopalatine ganglion and its role in various pain syndromes: From anatomy to clinical practice. Pain Pract..

[14] Mojica J, Mo B, Ng A (2017). Sphenopalatine ganglion block in the management of chronic headaches. Curr Pain Headache Rep..

[15] Robbins MS, Robertson CE, Kaplan E, Ailani J, Charleston L, Kuruvilla D, Blumenfeld A, Berliner R, Rosen NL, Duarte R, Vidwan J, Halker RB, Gill N, Ashkenazi A (2016). The sphenopalatine ganglion: Anatomy, pathophysiology, and therapeutic targeting in headache. Headache.

[16] Wang W, Lo ACY (2018). Diabetic retinopathy: Pathophysiology and treatments. Int. J. Mol. Sci..

[17] Duker JS, Belmont JB, Benson WE, Brooks HL, Brown GC, Federman JL, Fischer DH, Tasman WS (1991). Inadvertent globe perforation during retrobulbar and peribulbar anesthesia. Patient characteristics, surgical management, and visual outcome. Ophthalmology.

[18] Luchetti M, Magni G, Marraro G (2000). A prospective randomized double-blinded controlled study of ropivacaine 0.75% versus bupivacaine 0.5%-mepivacaine 2% for peribulbar anesthesia. Reg. Anesth. Pain Med..

[19] Gallagher EJ, Liebman M, Bijur PE (2001). Prospective validation of clinically important changes in pain severity measured on a visual analog scale. Ann. Emerg. Med..

[20] Friberg TR, Venkatesh S (1995). Alteration of pulse configuration affects the pain response during diode laser photocoagulation. Lasers Surg. Med..

[21] Chiu HH, Wu PC (2011). Manual acupuncture for relieving pain associated with panretinal photocoagulation. J. Altern. Complement Med..

[22] Yarnitsky D, Goor-Aryeh I, Bajwa ZH, Ransil BI, Cutrer FM, Sottile A, Burstein R (2003). 2003 Wolff Award: Possible parasympathetic contributions to peripheral and central sensitization during migraine. Headache.

[23] Peters GL (2019). Migraine overview and summary of current and emerging treatment options. Am. J. Manage Care.

[24] Khan S, Schoenen J, Ashina M (2014). Sphenopalatine ganglion neuromodulation in migraine: What is the rationale?. Cephalalgia.

[25] Yoo YJ, Yang HK, Choi JY, Kim JS, Hwang JM (2020). Neuro-ophthalmologic findings in visual snow syndrome. J. Clin. Neurol..

[26] Liu GT, Galetta SL (2001). The neuro-ophthalmologic examination (including coma). Ophthalmol. Clin. N. Am..

[27] Bloom SM, Brucker AJ (1997). Laser Surgery of the Posterior Segment.

[28] Binfalah M, Alghawi E, Shosha E, Alhilly A, Bakhiet M (2018). Sphenopalatine ganglion block for the treatment of acute migraine headache. Pain Res. Treat..

[29] Maguire MG, Liu D, Glassman AR, Jampol LM, Johnson CA, Baker CW, Bressler NM, Gardner TW, Pieramici D, Stockdale CR, Sun JK (2020). Visual field changes over 5 years in patients treated with panretinal photocoagulation or ranibizumab for proliferative diabetic retinopathy. JAMA Ophthalmol..

[30] Kim DH, Kang H, Hwang SH (2019). The effect of sphenopalatine block on the postoperative pain of endoscopic sinus surgery: A meta-analysis. Otolaryngol. Head Neck Surg..

[31] Wang P (2021). The efficacy of sphenopalatine ganglion block for pain management after endoscopic sinus surgery: A meta-analysis of randomized controlled studies. Eur. Arch. Otorhinolaryngol..

[32] Mansour SE, Browning DJ, Wong K, Flynn HW, Bhavsar AR (2020). The evolving treatment of diabetic retinopathy. Clin. Ophthalmol..

